# Supplementary material for: Identification and Profiling Analysis of microRNAs in Guava Fruit (Psidium guajava L.) and Their Role during Ripening
Source: Genes (Basel). 2023 Oct 31;14(11):2029. doi: 10.3390/genes14112029 (PMC10670931; doi:10.3390/genes14112029)
Supplement: Supplementary file 1 [file genes-14-02029-s001.zip › Supplementary material S1.pdf]

Supplementary data S1

Table S1. Mature miRNA sequences found in guava fruit

miRNA	Sequence	Length	Match Type
MIR10219	GAGGTTGTTTGGCTTGAGC	19	M 3' sub
MIR156a	TTGACAGAAGATAGAGAGCACT	22	M 5' super
MIR156a//MIR156b//MIR156c	TTGACAGAAGATAGAGAGCAC	21	M 5'
MIR159	TTTGGATTGAAGGGAGCTCTA	21	M 3'
	TTTTGGATTGAAGGGAGCTCT	21	M 3' sub/super
	TTTTTGGATTGAAGGGAGCTC	21	M 3' sub/super
	TTGGATTGAAGGGAGCTCTA	20	M 3' sub
	TTTGGATTGAAGGGAGCTCTAT	22	M 3' super
	TTTTGGATTGAAGGGAGCTCTA	22	M 3' super
	TGGATTGAAGGGAGCTCTA	19	M 3' sub
	AGCTGCTGGTCTATGGATCCC	21	M 5'
	AGCTGCTGGTCTATGGATC	19	M 5' sub
	CTTGCATATGCCAGGAGCTTC	21	M 3'
	TTTTGGATTGAAGGGAGCTC	20	M 3' sub/super
	AGCTGCTGGTCTATGGATCC	20	M 5' sub
	TTTTTGGATTGAAGGGAGCTCT	22	M 3' sub/super
	TTGCATATGCCAGGAGCTTC	20	M 3' sub
	TGAGCTGCTGGTCTATGGATC	21	M 5' sub/super
MIR159a	TTTGGATTGAAGGGAGCTCTAC	22	M 3' super
MIR159b	TTTGGATTGAAGGGAGCTCT	20	M 5' sub
	TTTGGATTGAAGGGAGCTCTT	21	M 3'
	TTTGGATTGAAGGGAGCTC	19	M 5' sub
	TTTGGATTGAAGGGAGCT	18	M 5' sub
	TTTGGATTGAAGGGAGCTCTC	21	M 3'
	TTTGGATTGAAGGGAGCTCTG	21	M 5'
	TTGGATTGAAGGGAGCTCT	19	M 5' sub
	ATTGGATTGAAGGGAGCTCT	20	M 3' sub
	TTTGGATTGAAGGGAGCTCTTC	22	M 3' super
	TTGGATTGAAGGGAGCTC	18	M 5' sub
	TTTGGATTGAAGGGAGCTCTCT	22	M 3' super
	TGGATTGAAGGGAGCTCT	18	M 5' sub
MIR159c	TTTGGATTGAAGGGAGCTCC	20	M 3' sub
MIR160c	TGCCTGGCTCCCTGTATGCT	20	M 5' sub
MIR162	TCGATAAACCTCTGCATCCAG	21	M 3'
MIR164a//MIR164b	TGGAGAAGCAGGGCACGTGCA	21	M 5'
MIR165a	TCGGACCAGGCTTCATCCCCCC	22	M 3' super
MIR165a//MIR165b	TCGGACCAGGCTTCATCCCCC	21	M 3'

	TCGGACCAGGCTTCATCCCC	20	M 3'
	TCGGACCAGGCTTCATCCC	19	M 3' sub
	TCGGACCAGGCTTCATCC	18	M 3' sub
MIR166	GGAATGTTGTCTGGCTCGAGG	21	M 5'
	TCGGACCAGGCTTCATTCCCCC	22	M 5'
	TTCGGACCAGGCTTCATTCC	20	M 3' sub
	TTTCGGACCAGGCTTCATTCC	21	M 3' sub
	GGAATGTTGTCTGGCTCGAGGC	22	M 5' super
	ATTTCGGACCAGGCTTCATTC	21	M 3' sub
	GGAATGTTGTCTGGCTCGAG	20	M 5' sub
	CGGACCAGGCTTCATTCCCCC	21	M 5' sub
	GGGAATGTTGTCTGGCTCGAGG	22	M 5' super
	GAATGTTGTCTGGCTCGAGGC	21	M 5' sub/super
	GGGAATGTTGTCTGGCTCGAG	21	M 5' sub/super
	CTCGGACCAGGCTTCATTCCCC	22	M 3' super
MIR166a	CTTCGGACCAGGCTTCATTCC	21	M 3' sub/super
	TTCGGACCAGGCTTCATTCCCC	22	M 3' super
	CCTCGGACCAGGCTTCATTCC	21	M 3' super
MIR166b	TCGGACCAGGCTTCATTCTTC	21	M 3' sub/super
	TCTCGGACCAGGCTTCATTCT	21	M 3'
MIR166c	GGAATGTTGTCTGGTTCGAGA	21	M 5' sub/super
	GGAATGTTGTCTGGTTCAAGG	21	M 5'
	GGAATGTTGTCTGGTCCGAGA	21	M 5' super
	TCGGACCAGGCTTCATTCCTC	21	M 3'
MIR166d	TATCGGACCAGGCTTCATTCC	21	M 3' sub/super
	ATCGGACCAGGCTTCATTCC	20	M 3' sub/super
MIR166e	TCTCGGACCAGGCTTCATTCCC	22	M 3' super
	TCTCGAACCAGGCTTCATTCC	21	M 3' sub/super
	CTCGGACCAGGCTTCATTCCC	21	M 3'
	TCGAACCAGGCTTCATTCCCC	21	M 3'
MIR166f	TCTCGGACCAGGCTTCATTCC	21	M 3'
	TCTCGGACCAGGCTTCATCCC	21	M 3' sub/super
	TCTCGGACCAGGCTTCATT	19	M 3' sub
	TCTCGGACCAGGCTTCATCC	20	M 3' sub/super
	CTCTCGGACCAGGCTTCATTCC	22	M 3' sub/super
	TCTCGGACCAGGCTTCATTCCT	22	M 3' super
MIR166h	TCTCGGACCAGGCTTCAATCC	21	M 3' sub/super
MIR166i	TTGGACCAGGCTTCATTCCCC	21	M 3'
	TCTCGGATCAGGCTTCATTCC	21	M 3' sub/super
	TTGGACCAGGCTTCATTCC	19	M 3' sub
	TCGGACCAGGCTTCATTCTC	20	M 3'

MIR166i//MIR166k	TCTTGGACCAGGCTTCATTCC	21	M 3'
MIR166m	GCGGACCAGGCTTCATTCCCC	21	M 3' super
MIR166p	TTCGGACCAGGCTTCATTCCC	21	M 3' super
	TCTCGGACCAGGCTCCATTCC	21	M 3' sub/super
MIR166u	TCTCGGACCAGGCTTCATTC	20	M 3'
MIR167a	TGAAGCTGCCAGCATGATCTA	21	M 5'
	TGAAGCTGCCAGCATGATCTGA	22	M 5'
	TGAAGCTGCCAGCATGATCTC	21	M 5'
	AAGCTGCCAGCATGATCTA	19	M 5' sub
MIR167b	TGAAGCTGCCAGCATGATCTGG	22	M 5'
MIR167b//MIR167f	AAGCTGCCAGCATGATCTAG	20	M 5' sub/super
MIR167c	TGAAGCTGCCAGCGTGATCTCA	22	M 5'
	TGAAGCTGCCAGCATGATCTT	21	M 5'
MIR167f	TGAAGCTGCCAGCATGATCTGC	22	M 5'
MIR168	TCGCTTGGTGCAGGTCGGGA	20	M 5'
	CGCTTGGTGCAGGTCGGGAAC	21	M 5' sub
	TCGCTTGGTGCAGGTCGGGAAC	22	M 5'
	TCGCTTGGTGCAGATCGGGAC	21	M 5'
MIR168a//MIR168b	TCGCTTGGTGCAGGTCGGGAC	21	M 5'
	TCGCTTGGTGCAGGTCGGGAA	21	M 5'
	CGCTTGGTGCAGGTCGGGAC	20	M 5' sub
	TCGCTTGGTGCAGGTCGGGACC	22	M 5' super
	GCTTGGTGCAGGTCGGGAC	19	M 5' sub
	CGCTTGGTGCAGGTCGGGACC	21	M 5' sub/super
	CTTGGTGCAGGTCGGGAC	18	M 5' sub
MIR168b	TCGCTTGGTGCAGGTCGGGTC	21	M 5'
MIR169a	CAGCCAAGGATGACTTGCCGG	21	M 5'
MIR169g	AAGCCAAGGATGAATTGCCGG	21	M 5'
MIR171f	TATTGGCCTGGTTCACTCAGA	21	M 5'
MIR2111	TAATCTGCATCCTGAGGTTTG	21	M 5'
MIR319	ATTGGACTGAAGGGAGCTCC	20	M 3'
MIR319a/MIR319b//MIR319c	TTGGACTGAAGGGAGCTCC	19	M 3' sub
	TTGGACTGAAGGGAGCTC	18	M 3' sub
MIR319a//MIR319c	TTGGACTGAAGGGAGCTCCC	20	M 3'
MIR319b	TTGGACTGAAGGGAGCTCCT	20	M 3'
	TGGACTGAAGGGAGCTCCC	19	M 3' sub
MIR319c	TTGGACTGAAGGGAGCTCCTT	21	M 3'
	TGGACTGAAGGGAGCTCCTT	20	M 3' sub
	TTGGACTGAAGGGAGCTCCTC	21	M 3' super
	TGGACTGAAGGGAGCTCCT	19	M 3' sub
	TGGACTGAAGGGAGCTCCTTT	21	M 3' sub/super

	TGGACTGAAGGGAGCTCCTC	20	M 3' sub/super
	CTTGGACTGAAGGGAGCTCCTT	22	M 3' super
MIR319q	TGGACTGAAGGGAGCTCCTTC	21	M 3'
MIR393	TCCAAAGGGATCGCATTGATCC	22	M 5'
	TCCAAAGGGATCGCATTGAT	20	M 5' sub
MIR393a	TCCAAAGGGATCGCATTGATT	21	M 5' sub
MIR393a//MIR393b	TCCAAAGGGATCGCATTGATC	21	M 5'
MIR393b	ATCATGCGATCCCTTCGGAAT	21	M 3' sub/super
MIR393c//MIR393d	TCCAAAGGGATCGCATTGATCT	22	M 5'
MIR394	TTGGCATTCTGTCCACCTCC	20	M 5'
MIR395	ATGAAGTGTTTGGGGGAACTC	21	M 3'
	ATGAAGTGTTTGGGGGAACT	20	M 3' sub
MIR395a	TGAAGTGTTTGGGGGAACTCC	21	M 5' sub
MIR396	AAGCTCAAGAAAGCTGTGGGA	21	M 3'
MIR396b	TTCCACAGCTTTCTTGAACTT	21	M 5'
	TTCAAGCTAGCTGTGGGAAG	20	M 3' sub
	GTTCAAGCTAGCTGTGGGAAG	21	M 3'
	TTCCACAGCTTTCTTGAACTG	21	M 5'
	TTCCACAGCTTTCTTGAACTTT	22	M 5' super
MIR397a	TCATTGAGTGCAGCGTTGATG	21	M 5'
	TCATTGAGTGCAGCGTTGAT	20	M 5'
	CATTGAGTGCAGCGTTGATGA	21	M 5' sub/super
	TCATTGAGTGCAGCGTTGA	19	M 5' sub
	TCATTGAGTGCAGCGTTGATGA	22	M 5' super
MIR398a	TGTGTTCTCAGGTCGCCCCTG	21	M 3'
	TGTGTTCTCAGGTCGCCC	18	M 3' sub
	TGTGTTCTCAGGTCGCCCCT	20	M 3' sub
MIR399e	CGCCAAAGGAGAGTTGCCCTC	21	M 3'
MIR482a	TCTTGCCAATACCACCCATGCC	22	M 3'
	TCTTGCCAATACCACCCATGC	21	M 3' sub
MIR482b	GGAATGGGCGGTTTGGGAAA	20	M 5' sub/super
	GGAATGGGCGGTTTGGGAA	19	M 5' sub/super
	TTTCCTATTCCTCCCATTCCAT	22	M 3'
	AATGGGCGGTTTGGGAAA	18	M 5' sub
	GAATGGGCGGTTTGGGAAA	19	M 5' sub
	GAATGGGCGGTTTGGGAA	18	M 5' sub
MIR482c	TTCCCAAGGCCGCCCATTCCGA	22	M 3'
	TTCCCAAGGCCGCCCATTCC	20	M 3' sub
	CCCAAGGCCGCCCATTCCGAC	21	M 3' sub/super
	TTCCCAAGGCCGCCCATTC	19	M 3' sub
	TCCCAAGGCCGCCCATTCCGAC	22	M 3' sub/super

MIR5139	AACCTGGCTCTGATACCA	18	M 5' sub
	AAACCTGGCTCTGATACCA	19	M 5'
MIR5139a	GAAACCTGGCTCTGATACCA	20	M 5'
MIR535a//MIR535b	TGACAACGAGAGAGAGCACGC	21	M 5'
MIR535d	TGACGACGAGAGAGAGCACGC	21	M 5'
	TGACGACGAGAGAGAGCACG	20	M 5' sub
MIR6300	GTCGTTGTAGTATAGTGG	18	M 3'
	GTCGTTGTAGTATAGTGGTA	20	M 3' super
MIR6478	CCGACCTTAGCTCAGTTGG	19	M 5' sub
	CCGACCTTAGCTCAGTTGGT	20	M 5' sub
MIR8155	TAACCTGGCTCTGATACCA	19	M 3'
MIR858a	TTCGTTGTCTGTTCGACC	18	M 5' sub
	TTCGTTGTCTGTTCGACCT	19	M 5' sub
	TTCGTTGTCTGTTCGACCTTG	21	M 5' sub/super

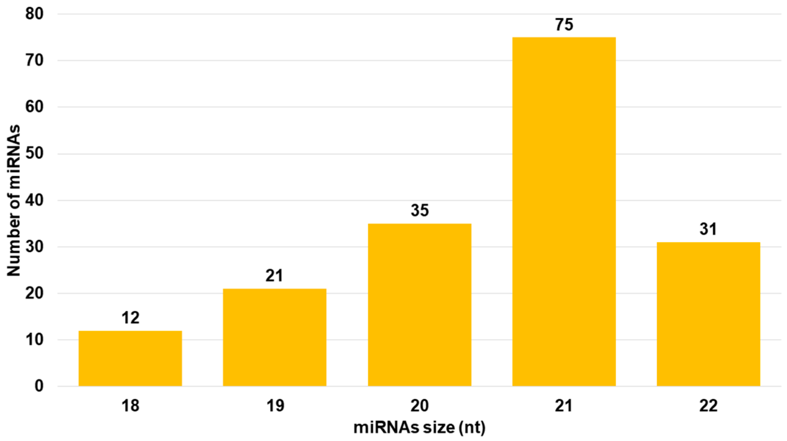

Supplementary Figure S1. Frequency distribution of sizes of miRNAs found in guava fruit.
